# Anxiety and depression in the post-pandemic era: concerns about viral mutation and re-outbreak

**DOI:** 10.1186/s12888-022-04307-1

**Published:** 2022-11-03

**Authors:** Zedong Li, Jin Li, Yamin Li, Feng Tian, Jin Huang, Zhihong Wang, Mingming Wang

**Affiliations:** 1 Clinical Nursing Teaching and Research Section, The Second Xiangya Hospital, Central South University, Changsha, China; 2 Department of Gastrointestinal Surgery, West China Hospital, Sichuan University, Chengdu, Sichuan Province China; 3 Department of Anesthesia, the Third Xiangya Hospital of Central South University, Changsha, China; 4 Department of Nursing, Zhangjiajie People’s Hospital, 192 Guyong road, Zhangjiajie, Hunan China

**Keywords:** Anxiety, Depression, COVID-19

## Abstract

**Background:**

The 2020 coronavirus pandemic (COVID-19) has been raging for more than 20 months, putting significant strain on public health systems around the world. Despite the fact that the pandemic has been effectively managed in certain countries, regional outbreaks and viral mutations continue to pose a threat to people's lives. The likelihood of post-pandemic changes in people's psychological situations warrants more investigation.

**Design and participants:**

This study was conducted in the context of another outbreak in Zhangjiajie, China, respondents (infected patients, healthy population) were required to complete self-administered questions and standardized questionnaires, including the patient health questionnaire-9 (PHQ-9), the generalized anxiety disorder-7 (GAD-7), and the Brief Illness Perception Questionnaire (BIPQ).

**Measures:**

We conducted an anonymous questionnaire survey of infected patients (excluding critically ill patients) in the confirmed COVID-19 ward of Zhangjiajie City People's Hospital's East Hospital from August 14 to 24, 2021, and used convenience sampling to survey medical staff and the general public to assess the psychological reactions of different populations during the delta variant outbreak pandemic. Differences in anxiety and depression severity were compared between groups, with logistic regression models constructed to explore potential factors associated with scoring clinical significant levels of depression and/or anxiety.

**Results:**

There is no significant difference (*p* value = 0.228) between anxiety and depression in patients (*n* = 53), general public (*n* = 97), medical personnel (*n* = 103), and support workers (*n* = 65). Females reported higher scores on the GAD-7 and the BIPQ, reduced communication with family and friends appeared to be a risk factor for clinically significant anxiety and depression.

**Conclusions:**

There were no significant differences in anxiety and depression across populations explored in this study, but females had higher anxiety and illness perception than males, and effective communication may help improve mental health.

**Supplementary Information:**

The online version contains supplementary material available at 10.1186/s12888-022-04307-1.

## Introduction

COVID-19 outbreaks began in late December 2019, posing a serious threat to public health and a significant challenge to economic development as well as social function in China and around the world [1]. Due to government regulation and public collaboration, new cases in China are predicted to be low in 2021, but scattered cases and small outbreaks will still persist. From September 1, 2021, more than 120,000 cases of COVID-19 had been confirmed in China, with more than 5,600 deaths. The COVID-19 pandemic has impacted every aspect of people's lives; moreover, COVID-19's long-term dominance has had a significant negative impact on the mental health of the people. [2–4].

In the early stages of the COVID-19 outbreak, healthy people, including the general public, pregnant women, the elderly, students, and even medical personnel, were found to have psychological illnesses [5–9]. During the pandemic, 20.9% of people with previous mental health difficulties were reported to have worsened symptoms[10]. A study reported that 37.5% of people with eating disorders experienced worsening symptoms and 56.2% exhibited other anxiety symptoms[11]. A study of psychological problems in parents of children hospitalized during a pandemic showed that they had significantly higher anxiety, depression, and dream anxiety scores compared with non-pandemic periods[12].

Quarantine, loss of income, frequent use of social media, shortages of vital supplies, social isolation, school closures, and other factors can all lead to psychological issues [13, 14]. Women are a population vulnerable to the COVID-19 pandemic[15], living alone[16], low or high educational attainment[17, 18], mental illness and substance abuse, and a history of other medical conditions may increase anxiety and/or depression during the pandemic [15, 19]. In addition, there are also some risk factors that appear to be inconsistently reported, such as age, with 31–40 year olds having a higher chance of developing depression[20], while another study claims there is no association between age and depression[17]. Anxiety, stress, fear, trauma, helplessness, and other psychological concerns should be considered and effectively addressed during the pandemic [21], therefore further research to elucidate potential risk factors in light of future outbreaks is needed.

COVID-19 transmission lasted longer and is more difficult to contain than the 2003 severe acute respiratory syndrome (SARS) outbreak. Despite the multiple efforts have been taken to prevent COVID-19 from spreading widely, localized outbreaks still occur after a long period with low increase rate of new confirmed case. The psychological impact of localized re-outbreaks on residents' mental health is also a source of concern.

A concert resulted a limited outbreak in July 2021, Zhangjiajie city, China, with more than 70 cases causing concern. Zhangjiajie city’s government decided to temporarily close all scenic spots. Unlike previous outbreaks in other parts of China, where just a few people were infected. This outbreak, in Zhangjiajie, had a larger number of infections, all of which were COVID-19 variant B.1.617.2 (delta). The first delta variation was discovered in India [22]. Up to date, new evidences suggest that patients with the delta version are more likely to admitted to the hospital than those with the alpha variant [23]. The B.1.617.2 (delta) type, which has resulted in the highest number of infections in China since the delta variant's discovery. Herein, it's important investigating whether the emergence of new viral subtypes changes people’s perspectives of COVID-19.

In the early stage of COVID-19 pandemic, healthcare practitioners around the world are likely to be under a lot of pressure to work, which may lead to mental health issues [24]. The pandemic not only harms physical health, but it also exacerbates psychological issues, possibly as a result of COVID-19 altering how people socialize, work, study, and live [25].

Emerging public health events put people's physical and mental health at risk. Some people who experience a pandemic will develop stress-related symptoms. These symptoms may disappear due to self-healing, or they may be followed by post-traumatic stress disorder[26, 27], so those at greatest risk need to be identified. The purpose of this study was to investigate whether localized re-outbreak have an impact on people's mental health, particularly anxiety and depression, and to explore high-risk factors.

## Materials and methods

### Participants

From August 14 to 24, 2021, we conducted an anonymous questionnaire survey of infected patients (excluding critically ill patients) in the confirmed New Crown Pneumonia ward of the East Hospital of Zhangjiajie City People's Hospital, and used convenience sampling to survey medical staff and the normal population to assess the psychological reactions of different populations during the delta variant outbreak pandemic. A total of 54 responses were collected from confirmed patients, and 266 were collected from the healthy people. After excluding two noncompliant questionnaires, 318 valid questionnaires (99.3% effective rate) remained.

### Survey methods

The survey was anonymously self-administered at www.wjx.cn, each entry had to be completed before it can be submitted, and each IP address only submit one response. Members of the study team distributed the questionnaire via WeChat groups and encouraged participants to share it with their friends, these WeChat groups contained individuals from the general public, medical and support staff groups. The purpose and significance of the survey were introduced by using uniform, standardized guidelines in the qustionnaire. The survey of infected patients was completed by the ward nurses. The link to the questionnaire was provided by the nurse during the patient's free time and was completed voluntarily with the help of the nurse.

### Study instruments

#### Self-prepared general information questionnaire

Gender, age, and other factors are included in the questionnaire.

Are you a member of the medical team that assists Zhangjiajie city?

Have you been infected by COVID-19?

Are any of your acquaintances affected by COVID-19?

Have you ever been forced to live alone for more than two weeks due to an outbreak?

Do you feel worried and uneasy as a result of the outbreak's restricted interaction and conversation with individuals close to you?

Has the outbreak had a substantial financial impact on you and your family?

In addition, 12 questions about COVID-19 attitudes were included in the questionnaire. We also included an open-ended fill-in-the-blank question: What is your main concern regarding the pandemic re-outbreak in the region and the expansion of the Delta subtype? All questionnaires can be found in supplementary material 1.

#### Scale for anxiety and depression

In previous studies, the Generalized Anxiety Disorder 7 (GAD-7) scale has shown to be a reliable instrument for identifying probable instances of generalized anxiety disorder. GAD-7 scores were split into four groups based on the scale: 0–5, 6–9, 10–14, and 15–21, which corresponded to none, mild, moderate, and severe anxiety, respectively [28]. In this investigation, the scale's Cronbach's coefficient was 0.923.

The Patient Health Questionnaire-9 (PHQ-9) is a highly sensitive measure for changes in depressive symptoms that evaluates nine depression factors. PHQ-9 scores were split into five categories based on the scale: 0–4, 5–9, 10–14, 15–19, and 20–27, which corresponded to none, mild, moderately severe, and severe depression, respectively [29, 30]. In this investigation, the scale's Cronbach's coefficient was 0.916.

A total score of 10 on the PHQ-9 suggests likely depression with a sensitivity of 80% and specificity of 92 percent [31, 32], and a total score of 10 on the GAD-7 indicates possible anxiety with a sensitivity of 89 percent and specificity of 82 percent [33–35].

A total score of ≥ 10 on the GAD-7 indicates possible anxiety and a total score of ≥ 10 on the PHQ-9 indicates possible depression[36]. Despite the fact that GAD-7 and PHQ-9 score were splitted into four groups, we treated them as continuous for the primary analysis.

#### Patients’ perception of the disease

The Brief Illness Perception Questionnaire (BIPQ was used to evaluate disease perception in infected patients) is a unidimensional questionnaire that investigates patients' perceptions of illness, with 9 items, 8 of which were scored on a scale of 0 to 10 on 11 levels, and 1 open-ended question to explore the causal relationship of illness[37]. The total possible score range was 0–80, with higher scores indicating more severe negative perceptions of the disease by the patient. The Cronbach′s α coefficient was 0.673. Because the patients were all infected, causality was not explored here.

#### Open-ended fill-in-the-blank question

To understand the concerns of infected people, we designed an open-ended fill-in-the-blank question. The frequency of words in the answers was counted and a word cloud was created using the wordcloud2 R package, with word frequency represented by font size.

### Statistical analysis

R (version 4.0.5) was used to analyze the data, comparison of PHQ and GAD scores between virus situation, group were conducted with the non-parametric Kruskal–Wallis test, comparison of PHQ and GAD scores between gender, isolation, friend infection, unsettling, economic difficulties were conducted with the non-parametric Wilcoxon test, comparison of PHQ, BIPQ score between gender, virus situation were conducted with the non-parametric Wilcoxon test. Logistic regression analysis was used to identify risk factors associated with scoring in the range of clinically significant anxiety and depression. Pearson correlations analysis was used to evaluate the correlation of scores. A *P* value < 0.05 indicates statistical significance.

## Results

### Basic information about the study population

A total of 168 medical personnel participated in the study, 65 medical staff who supported Zhangjiajie, 53 patients, 11 of whom had asymptomatic infections, and 97 other healthy people (non-medical personnel). Table 1 shows the characteristics of the respondents. 11 asymptomatic carriers, 42 infections and symptoms, and 265 not infected were included in our study.

**Table 1:** Demographics of the respondents

	**Not infected**	**Asymptomatic carrier**	**Infection and symptoms**
	**(*N* = 265)**	**(*N* = 11)**	**(*N* = 42)**
**Gender**			
Male	48 (18.1%)	5 (45.5%)	17 (40.5%)
Female	217 (81.9%)	6 (54.5%)	25 (59.5%)
**Friend Infection**			
no	263 (99.2%)	9 (81.8%)	20 (47.6%)
yes	2 (0.8%)	2 (18.2%)	22 (52.4%)
**Isolation**			
no	228 (86.0%)	6 (54.5%)	28 (66.7%)
yes	37 (14.0%)	5 (45.5%)	14 (33.3%)
**GAD-7 score**			
Mean (SD)	3.62 (3.83)	3.09 (2.59)	2.90 (4.30)
Median [Min, Max]	2.00 [0, 21.0]	3.00 [0, 7.00]	1.00 [0, 18.0]
**GAD-7 stage**			
none	175 (66.0%)	8 (72.7%)	33 (78.6%)
mild	71 (26.8%)	3 (27.3%)	5 (11.9%)
moderate	14 (5.3%)	0 (0%)	3 (7.1%)
severe	5 (1.9%)	0 (0%)	1 (2.4%)
**PHQ-9 score**			
Mean (SD)	4.71 (4.66)	2.00 (2.90)	4.60 (5.89)
Median [Min, Max]	4.00 [0, 27.0]	1.00 [0, 9.00]	2.00 [0, 24.0]
**PHQ-9 stage**			
None	139 (52.5%)	9 (81.8%)	26 (61.9%)
Slight	97 (36.6%)	2 (18.2%)	8 (19.0%)
Moderate	19 (7.2%)	0 (0%)	5 (11.9%)
Moderately Severe	7 (2.6%)	0 (0%)	1 (2.4%)
Severe	3 (1.1%)	0 (0%)	2 (4.8%)
**Group**			
patient	0 (0%)	11 (100%)	42 (100%)
medical personnel	103 (38.9%)	0 (0%)	0 (0%)
support staff	65 (24.5%)	0 (0%)	0 (0%)
others	97 (36.6%)	0 (0%)	0 (0%)

### Differences in GAD-7 score and PHQ-9 score between subgroups

In different virus situation, mean scores for asymptomatic carrier (*n* = 11) were 3.09 (SD = 2.59) on the GAD-7 scale, and 2.00 (SD = 2.90) on the PHQ-9 scale, mean scores for people with infection and symptoms (*n* = 42) were 2.90 (SD = 4.3,) on the GAD-7 scale, and 4.60 (SD = 5.89) on the PHQ-9 scale. Mean scores for not infected people (*n* = 248) were 3.62 (SD = 3.83) on the GAD-7 scale, and 4.71 (SD = 4.66) on the PHQ-9 scale. Results from the Kruskal–Wallis test suggested that there was no difference in GAD-7 and PHQ-9 scores between different virus situations (*p* value = 0.0838).

GAD-7 score was statistically different (*p* value = 0.0231) between male (*N* = 70, mean = 2.90, SD = 4.28) and female (*N* = 248, mean = 3.67, SD = 3.72), females scored higher on GAD-7, but not PHQ-9 score.

It is worth noting that there is no significant difference in the GAD-7 and PHQ-9 scores of patients, general public, medical personnel, and support staff (*p* value = 0.228), which indicates that in this re-outbreak, working in the pandemic area will not increase the anxiety of medical personnel, and infection with the virus does not significantly increase the anxiety of patients.

Isolation (> 2 weeks) does not lead to anxiety and depression, but unsettling due to reduced communication with friends and family, virus infection of friends, and economic difficulties due to the pandemic will significantly increase GAD-7 and PHQ-9 scores (Table 2).

**Table 2:** Factors associated with anxiety and depression

	**GAD-7 score**			**PHQ-9 score**		
	**Mean (SD)**	**Median [Min, Max]**	***P* value**	**Mean (SD)**	**Median [Min, Max]**	***P* value**
**Virus situation**						
Asymptomatic carrier(*N* = 11)	3.09 (2.59)	3.00 [0, 7.00]	0.152	2.00 (2.90)	1.00 [0, 9.00]	0.0838
Infection and symptoms(*N* = 42)	2.90 (4.30)	1.00 [0, 18.0]		4.60 (5.89)	2.00 [0, 24.0]	
Not infected(*N* = 265)	3.62 (3.83)	2.00 [0, 21.0]		4.71 (4.66)	4.00 [0, 27.0]	
**Gender**						
Male(*N* = 70)	2.90 (4.28)	1.50 [0, 21.0]	0.0231	4.36 (5.50)	2.50 [0, 27.0]	0.249
Female(*N* = 248)	3.67 (3.72)	3.00 [0, 21.0]		4.67 (4.60)	4.00 [0, 24.0]	
**Group**						
medical personnel(*N* = 103)	3.42 (3.47)	3.00 [0, 14.0]	0.219	4.70 (4.56)	5.00 [0, 19.0]	0.228
others(*N* = 97)	3.45 (3.98)	2.00 [0, 21.0]		4.52 (5.01)	3.00 [0, 27.0]	
patient(*N* = 53)	2.94 (3.98)	1.00 [0, 18.0]		4.06 (5.48)	2.00 [0, 24.0]	
Support staff(*N* = 65)	4.17 (4.14)	3.00 [0, 20.0]		5.00 (4.32)	5.00 [0, 21.0]	
**Isolation**						
no(*N* = 262)	3.47 (3.64)	2.00 [0, 21.0]	0.52	4.48 (4.57)	4.00 [0, 24.0]	0.687
yes(*N* = 56)	3.68 (4.79)	2.00 [0, 21.0]		5.14 (5.79)	3.50 [0, 27.0]	
**Friend Infection**						
no(*N* = 292)	3.64 (3.94)	2.00 [0, 21.0]	0.0339	4.78 (4.86)	4.00 [0, 27.0]	0.00631
yes(*N* = 26)	1.96 (2.36)	1.00 [0, 7.00]		2.50 (3.64)	0.500 [0, 13.0]	
**Unsettling**						
no(*N* = 220)	2.83 (3.17)	2.00 [0, 14.0]	< 0.001	4.06 (4.18)	3.00 [0, 19.0]	0.016
yes(*N* = 98)	5.02 (4.75)	4.00 [0, 21.0]		5.81 (5.82)	4.00 [0, 27.0]	
**Economic difficulties**						
no(*N* = 198)	2.79 (3.25)	2.00 [0, 21.0]	< 0.001	3.91 (4.13)	3.00 [0, 21.0]	0.00336
yes(*N* = 120)	4.68 (4.47)	4.00 [0, 21.0]		5.73 (5.58)	5.00 [0, 27.0]	

Economic difficulties: Has the outbreak had a significant impact on your family's financial resources? Isolation: Have you ever lived alone for more than two weeks because of the outbreak? Unsettling: Do you feel disturbed and uncomfortable because of the reduced contact and communication with people close to you due to the outbreak?

*Abbreviations: PHQ-9* Patient health questionnaire-9, *GAD-7* Generalized anxiety disorder-7, *SD* Standard deviation

### Patient's perception of disease

The BIPQ was used to assess infected individuals' illness perception.

Table 3 shows that mean BIPQ scores for males (*n* = 22) were 46.5 (SD = 13.1) and females (*n* = 31) were 51.6 (SD = 11.6), indicating that there was a difference between male and female (*p* value = 0.045). Men, on the other hand, have a more positive attitude toward the disease. There was no difference in disease perception between symptomatic and asymptomatic patients (*p* value = 0.532).

**Table 3:** Factors associated with illness perception

	**BIPQ score**		
	**Mean (SD)**	**Median [Min, Max]**	***P* value**
**Gender**			
Male(*N* = 22)	46.5 (13.1)	43.5 [25.0, 75.0]	0.045
Female(*N* = 31)	51.6 (11.6)	52.0 [17.0, 74.0]	
**Virus_situation**			
Asymptomatic carrier(*N* = 11)	47.4 (8.49)	46.0 [31.0, 60.0]	0.532
Infection and symptoms(*N* = 42)	50.1 (13.2)	49.0 [17.0, 75.0]	

**Fig. 1:**
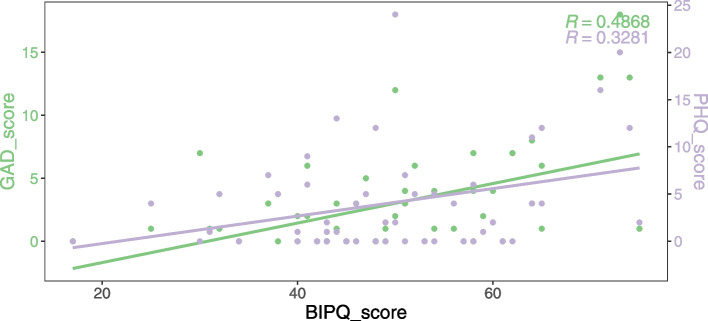
Correlation of BIPQ score with GAD score and PHQ score

Pearson correlations were utilized to evaluate the association between disease perception and anxiety and depression to learn more about it. The BIPQ score was shown to be favourably connected with both the GAD and PHQ scores, with correlation coefficients of 0.49 (*p* < 0.001) and 0.33 (*p* < 0.001), respectively (Fig. 1).

#### 12 COVID-19 related questions

We created twelve questions to study people's perspectives toward government strategies, virus mutation, vaccines, transmission, re-breakout, and other relevant topics. Answers Percentage of the 12 questions can be found in Table 4. COVID-19 transmission is mostly manageable, according to 40.8% of respondents. Regarding vaccines, 56.2 percent believe that COVID-19 can be mostly controlled using vaccines. The majority of people are hopeful about vaccinations' ability to battle the delta form; 42.4% and 36.7% of them believe it is mostly controlled and controllable, respectively. COVID-19-related deaths and injuries are mostly preventable, according to 54.7% of persons.

**Table 4:** COVID-19 related questions

	completely out of control	most of them can't control	basically controllable	Most can be controlled	full control
How do you think the transmition of COVID-19?	0.283018868	0.009433962	0.273584906	0.408805031	0.02515723
What do you think about the role of vaccines in the prevention and control of COVID-19?	0.267295597	0	0.119496855	0.562893082	0.05031447
What do you think is the role of vaccines in the prevention and control of COVID-19 subtype (delta)?	0.367924528	0.003144654	0.08490566	0.424528302	0.11949686
To what extent do you think it is possible to control deaths and injuries caused by COVID-19?	0.251572327	0.018867925	0.13836478	0.547169811	0.04402516
To what extent do you think the economic losses caused by COVID-19 can be controlled?	0.43081761	0.031446541	0.072327044	0.323899371	0.14150943
To what extent do you think national policies can control the COVID-19 epidemic?	0.22327044	0.003144654	0.311320755	0.433962264	0.02830189
	Basically conform	Fully compliant	Mostly conform	Mostly not	Not at all
COVID-19 will spread widely again	0.254716981	0.012578616	0.072327044	0.474842767	0.18553459
COVID-19 can mutate (e.g., Delta) and can be more difficult to control?	0.393081761	0.072327044	0.135220126	0.29245283	0.10691824
COVID-19 can cause significant illness and even death?	0.179245283	0.031446541	0.06918239	0.455974843	0.26415094
COVID-19 has an impact on your life?	0.336477987	0.238993711	0.169811321	0.176100629	0.07861635
COVID-19 can pose a significant threat to you and your family's health and lives?	0.248427673	0.103773585	0.13836478	0.308176101	0.20125786
COVID-19 can pose a great threat to your work and daily life order?	0.289308176	0.201257862	0.13836478	0.273584906	0.09748428

In terms of COVID-19-related economic impact, most people remain cautious; 43% and 32% of them believe it is largely controllable and mostly controllable, respectively. The majority of people are optimistic about China's contribution to disease prevention and control. 43.3% of people felt the outbreak is mostly under control, and 31.1% claimed it was completely under control due to government initiatives. The majority of Americans do not believe there will be another widespread outbreak, with 47.4% responding "mainly not." Some people are pessimistic about the Delta variant; 39.3% believe that the Delta type will be more difficult to handle. COVID-19 can cause serious disease and even death, according to 45.5% who replied "mainly not" and 26.4% who answered, "not at all." More than half of those polled indicated the outbreak has had an impact on their life, although the majority stated there are no severe health hazards (Table 4). More than half stated they were affected by work and order in their daily lives. The majority of people are hopeful about the pandemic, but many others claim that it has had an impact on their life and jobs.

### Logistic regression analysis of anxiety and depression

**Fig. 2:**
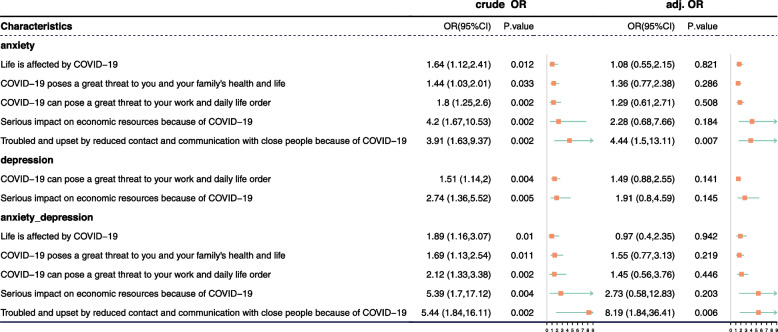
Logistic regression analysis of anxiety and depression

Logistic regression analysis was performed with the presence of depression/anxiety symptoms as the dependent variable (No = 0, Yes = 1) and gender, age, and answers to 12 COVID-19-related questions, including isolation, unsettling, and economic difficulties, people group, and virus situation as the independent variables. The results showed a higher the incidence of anxiety (adj. OR = 4.44; 95% CI, 1.5–13.11, *p* value = 0.007) and combined anxiety-depression (adj. OR = 8.19; 95% CI, 1.84–36,341, *p* value = 0.006) in the population who was troubled and upset by reduced contact and communication with close friends and relatives due to COVID-19 (Fig. 2).

### Concerns about the outbreak and delta variant

**Fig. 3:**
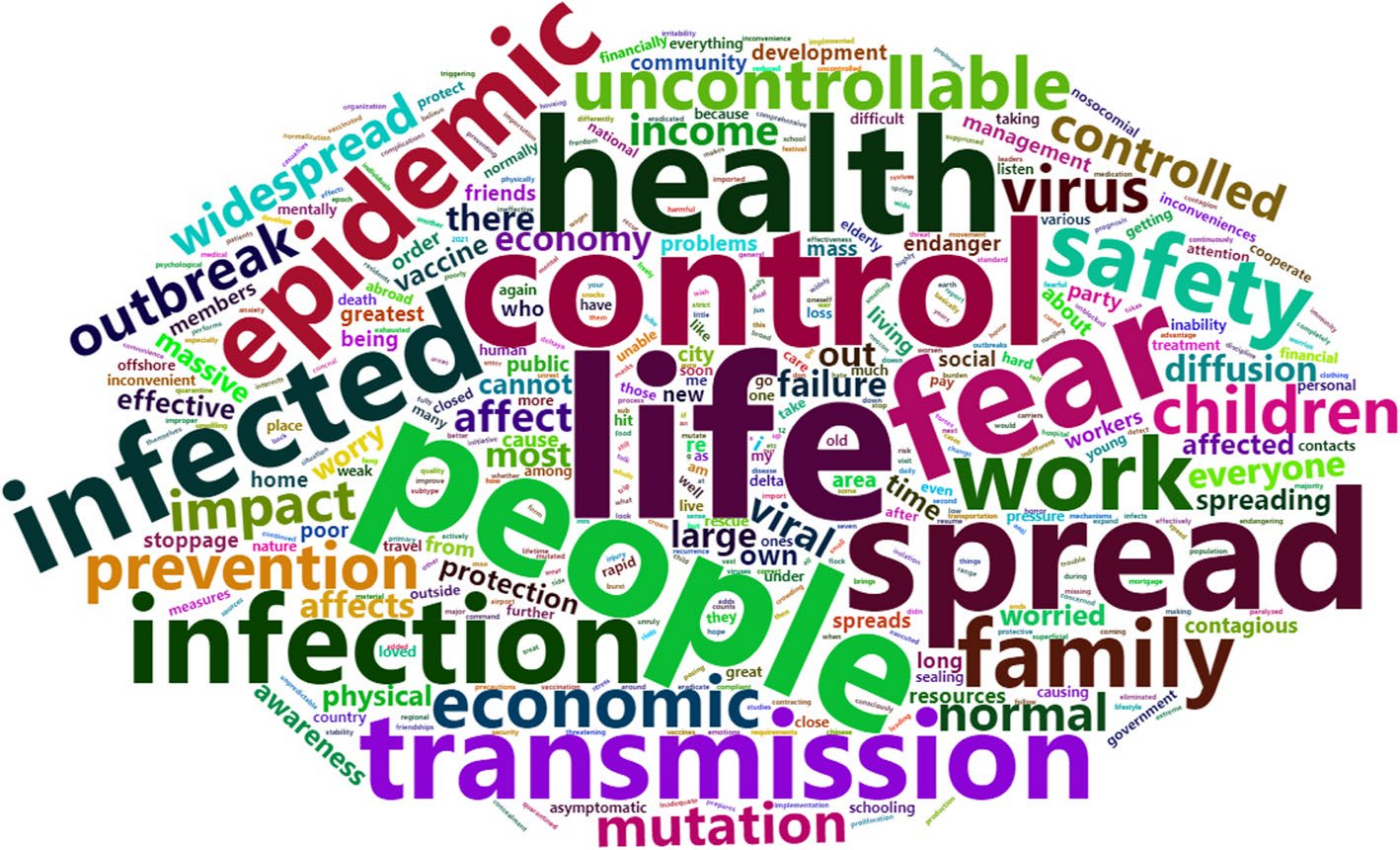
Word cloud of concerns about the outbreak and delta variant

Open-ended fill-in-the-blank question allows us to better understand the concerns of infected person. 87.4% expressed their concern (12.6% of participants left it blank), high-frequency terms were tallied and a word cloud was created using the wordcloud2 R package, and the word cloud of the text is shown in Fig. 3. Spread, control, infection, transmission, mutation, pandemic, outbreak, family, children, and other words appeared frequently. It also demonstrates that transmission, mutation, outbreak control, children, and families remain the primary issues.

Furthermore, some of the comments are deserving of our attention, such as:

"During the Spring Festival of 2021, another huge version spread."

"Some people do not record the trip and keep the outbreak hidden."

"A huge spread was induced by habituation, enormous populations, and inadequate protection."

"It's how the rest of the world acts that matters."

"After I recovered, people treated me differently, spoke about me, and it affected my children's education, friendships, and everything."

## Discussion

Emerging public health events, particularly emerging infectious diseases, are marked by their unpredictability and suddenness, as well as their widespread, prevalence of hazards, fatality rate, and complexity of the intervention, all of which impact people's physical health and safety, as well as negative emotions such as public panic and anxiety. COVID-19 is a novel infectious disease with high pathogenicity, a high infection rate, rapid transmission, and widespread spread. Most people who have been through a crisis suffer stress-related symptoms that go away on their own, but some people experience negative emotions such as sadness and anxiety, as well as posttraumatic stress disorder (PTSD), which will have serious and long-term consequences [38].

There were more reports on the psychological conditions of the people during the early stages of the pandemic, but few investigations have been performed on the psychological conditions of the population after the pandemic’s extended survival and localized re-outbreak. One and a half years after the first COVID-19 outbreak, we exploited this outbreak in Zhangjiajie to investigate the psychological state of the population in the limited outbreak area. Accoding to our findings, there are no significant differences in anxiety and depression scores between the general public, frontline medical personnel, general medical staff, and those infected with the delta variation. The prevalence of anxiety and depression among frontline medical personnel fighting novel coronavirus pneumonia in Gansu, China, reported in April 2020, was 11.4% (anxiety) and 45.6% (depression) for physicians and 27.9% (anxiety) and 43.0% (depression) for nurses, respectively. In this re-outbreak, the prevalence of anxiety and depression among frontline medical staff was much lower than previously documented [39]. Furthermore, Li et al*.* stated in June 2020 that frontline medical professionals were twice as likely to experience anxiety and depression as non frontline medical employees. This also contradicts our current findings, which indicated no significant difference in the prevalence of anxiety and depression among frontline medical professionals and non frontline medical staff [40]. This may be related to the fact that the average number of patients presenting to the clinic were less severe. In addition, the number of infections in this outbreak was lower compared to the initial outbreak. Widespread nucleic acid testing allows for earlier screening of infected individuals, the rollout of the vaccine has also helped tremendously in the control of COVID-19[41, 42]. Teleconnectivity was used in various aspects such as person-to-person communication, education, telehealth, etc. [43, 44], which may all also contribute to the reduced prevalence of anxiety and depression.

According to the results of Lu et al., females, gregarious persons, and critical workers, among others, experienced extreme anxiety when the lockdown was announced, but this worry subsided quickly afterward. Mental health deteriorated during the lockdown but improved following [45]. Eric et al. reported on depression and anxiety in the overall population of Hong Kong, between the 24^th^ of April and 3^rd^ of may, 2020 [36]. 88% and 80% of people in Zhangjiajie and Hong Kong fell into the slight to none category on the PHQ, respectively, and 93% of people in Zhangjiajie and 86% of people in Hong Kong had a GAD score < 10. These results appear to indicate that as the COVID-19 pandemic has progressed since the vaccine was introduced and public awareness of COVID-19 has increased, people are gradually adapting to coexist with the vrius, and the psychological situation is improving, even though vrius is still mutating and causing small outbreaks.

Females are more vulnerable to stress and PTSD than males [46]. According to this study, females have higher scores of depression and anxiety,, which is consistent with early findings in the COVID-19 pandemic [15, 47]. Furthermore, according to our findings, whether the pandemic resulted in less communication with loved ones and friends was a key contributor to despair and anxiety. During isolation treatment, patients may be encouraged to interact with family and friends through video phones to minimise anxiety.

People are concerned about the virus's spread and mutation, the health of their family members, and the financial troubles that some people are enduring as a result of the outbreak, which occurred more than a year and a half after the COVID-19 outbreak. Social stigma is a major source of worry. The negative link between a person or a group of individuals with certain features and a certain condition is referred to as social stigma [48]. This is common among frontline medical workers [39] and people recovering from infection [49], who may be stigmatized, discriminated against, treated differently, and have their professional life impacted, among other things. The government and the media should distribute effective scientific knowledge that will enable people infected with COVID-19 to destigmatize themselves, and thereby safeguard their mental health, as well as help people, comprehend COVID-19 and viral variations accurately.

There are some limitations of this study that need to be noted. Firstly, the impact of this outbreak was limited, with less than 100 patients infected, resulting in a tiny sample size. Secondly, as the COVID-19 can only collect data from self-reported scales, patience, attitude, and computer skill all influence questionnaire responses. Finally, the findings of this study may not be generalizable to future re-outbreak, because the scope of future re-outbreak and the number of people affected may have a different impact on the results.

Limitations should be considered when interpreting the results of this study. First, the number of people infected in this localized outbreak was limited, as was its impact on people's mental health, In the future, it is not clear whether COVID-19 will be more severe or when it will be eradicated, and it is possible that people's psychological status will vary at different times. In addition, the present study also suffers from selectivity bias in the sampling method, a small sample size, and the limited geographic area covered by the study. The findings in this study may not be generalizable to other populations.

In conclusion, there is no significantly difference in the mental health of patients, general public, medical personnel and support staff. Reduced communication with family and friends is a risk factor for anxiety and depression during the pandemic, and females in this sample appeared to report higher levels of anxiety and different illness perception than males.

## Supplementary Information

**Additional file 1. **Questionnaire.

## Data Availability

Mingming Wang confirm to have full access to all the data in the study, and to take responsibility for the integrity of the data and the accuracy of the data analysis. The datasets used and/or analysed during the current study available from the corresponding author (Mingming Wang) on reasonable request.
