# Supplementary material for: Anxiety and depression in the post-pandemic era: concerns about viral mutation and re-outbreak
Source: BMC Psychiatry. 2022 Nov 3;22:678. doi: 10.1186/s12888-022-04307-1 (PMC9630811; doi:10.1186/s12888-022-04307-1)
Supplement: Supplementary file 1 — Additional file 1. Questionnaire. [file 12888_2022_4307_MOESM1_ESM.docx]

**Questionnaire**

**Basic Information**

**1.Are you a member of the medical team that assists Zhangjiajie city?**

| ○Yes |
| --- |
| ○No |

**2.Gender**

| ○Male |
| --- |
| ○Female |

**3.Age**

| ○12-20 |
| --- |
| ○21-25 |
| ○26-35 |
| ○36-45 |
| ○46-55 |
| ○56+ |

**4.Have you been infected by COVID-19?**

| ○ No infection |
| --- |
| ○Asymptomatic carriers |
| ○ Infection and symptoms |
| ○ Once infected, now cured |

**5.Your area**

○Medium and high risk areas

○Low-risk areas

**6. Are any of your acquaintances affected by COVID-19?**

| ○Yes |  |
| --- | --- |
| ○No |  |

**7.Have you ever been forced to live alone for more than two weeks due to an outbreak?**

| ○Yes |  |
| --- | --- |
| ○No |  |

**8.Do you feel worried and uneasy as a result of the outbreak's restricted interaction and conversation with individuals close to you?**

| ○Yes |  |
| --- | --- |
| ○No |  |

**9.Has the outbreak had a substantial financial impact on you and your family?**

| ○Yes |  |
| --- | --- |
| ○No |  |

**12 questions about COVID-19 attitudes**

|  | completely out of control | most of them can't control | basically controllable | Most can be controlled | full control |
| --- | --- | --- | --- | --- | --- |
| How do you think the transmition of COVID-19? | ○ | ○ | ○ | ○ | ○ |
| What do you think about the role of vaccines in the prevention and control of COVID-19? | ○ | ○ | ○ | ○ | ○ |
| What do you think is the role of vaccines in the prevention and control of COVID-19 subtype (delta)? | ○ | ○ | ○ | ○ | ○ |
| To what extent do you think it is possible to control deaths and injuries caused by COVID-19? | ○ | ○ | ○ | ○ | ○ |
| To what extent do you think the economic losses caused by COVID-19 can be controlled? | ○ | ○ | ○ | ○ | ○ |
| To what extent do you think national policies can control the COVID-19 epidemic? | ○ | ○ | ○ | ○ | ○ |

|  | Basically conform | Fully compliant | Mostly conform | Mostly not | Not at all |
| --- | --- | --- | --- | --- | --- |
| COVID-19 will spread widely again? | ○ | ○ | ○ | ○ | ○ |
| COVID-19 can mutate (e.g., Delta) and can be more difficult to control? | ○ | ○ | ○ | ○ | ○ |
| COVID-19 can cause significant illness and even death? | ○ | ○ | ○ | ○ | ○ |
| COVID-19 has an impact on your life? | ○ | ○ | ○ | ○ | ○ |
| COVID-19 can pose a significant threat to you and your family's health and lives? | ○ | ○ | ○ | ○ | ○ |
| COVID-19 can pose a great threat to your work and daily life order? | ○ | ○ | ○ | ○ | ○ |

**What is your main concern regarding the pandemic re-outbreak in the region and the expansion of the Delta subtype?**

**_________________________________**

____________________________________________________________________

**GAD-7 (General Anxiety Disorder-7)**

1. **Feeling nervous, anxious, or on edge**

Not at all

Several days

More than half the days

Nearly every day

1. **Not being able to stop or control worrying**

Not at all

Several days

More than half the days

Nearly every day

1. **Worrying too much about different things**

Not at all

Several days

More than half the days

Nearly every day

1. **Trouble relaxing**

Not at all

Several days

More than half the days

Nearly every day

1. **Being so restless that it's hard to sit still**

Not at all

Several days

More than half the days

Nearly every day

1. **Becoming easily annoyed or irritable**

Not at all

Several days

More than half the days

Nearly every day

1. **Feeling afraid as if something awful might happen**

Not at all

Several days

More than half the days

Nearly every day

**PHQ-9 (Patient Health Questionnaire-9)**

1. **Little interest or pleasure in doing things?**

Not at all

Several days

More than half the days

Nearly every day

1. **Feeling down, depressed, or hopeless?**

Not at all

Several days

More than half the days

Nearly every day

1. **Trouble falling or staying asleep, or sleeping too much?**

Not at all

Several days

More than half the days

Nearly every day

1. **Feeling tired or having little energy?**

Not at all

Several days

More than half the days

Nearly every day

1. **Poor appetite or overeating?**

Not at all

Several days

More than half the days

Nearly every day

1. **Feeling bad about yourself — or that you are a failure or have let yourself or your family down?**

Not at all

Several days

More than half the days

Nearly every day

1. **Trouble concentrating on things, such as reading the newspaper or watching television?**

Not at all

Several days

More than half the days

Nearly every day

1. **Moving or speaking so slowly that other people could have noticed? Or so fidgety or restless that you have been moving a lot more than usual?**

Not at all

Several days

More than half the days

Nearly every day

1. **Thoughts that you would be better off dead, or thoughts of hurting yourself in some way?**

Not at all

Several days

More than half the days

Nearly every day

The Brief Illness Perception Questionnaire

For the following questions, please circle the number that best corresponds to your views:

| **How much does your illness affect your life?** | | | | | | | |  |  |
| --- | --- | --- | --- | --- | --- | --- | --- | --- | --- |
| 0 1 | 2 | 3 | 4 | 5 | 6 | 7 | 8 | 9 | 10 |
| no affect |  |  |  |  |  |  |  |  | severely |
| at all |  |  |  |  |  |  |  |  | affects my life |
| **How long do you think your illness will continue?** | | | | | | | |  |  |
| 0 1 | 2 | 3 | 4 | 5 | 6 | 7 | 8 | 9 | 10 |
| a very | | | | | | | |  | forever |
| short time | | | | | | | |  |  |
| **How much control do you feel you have over your illness?** | | | | | | | |  |  |
| 0 1 | 2 | 3 | 4 | 5 | 6 | 7 | 8 | 9 | 10 |
| absolutely |  |  |  |  |  |  |  |  | extreme amount |
| no control |  |  |  |  |  |  |  |  | of control |
| **How much do you think your treatment can help your illness?** | | | | | | | |  |  |
| 0 1 | 2 | 3 | 4 | 5 | 6 | 7 | 8 | 9 | 10 |
| not at all | | | | | | | |  | extremely |
|  | | | | | | | |  | helpful |
| **How much do you experience symptoms from your illness?** | | | | | | | |  |  |
| 0 1 | 2 | 3 | 4 | 5 | 6 | 7 | 8 | 9 | 10 |
| no symptoms |  |  |  |  |  |  |  |  | many severe |
| at all |  |  |  |  |  |  |  |  | symptoms |
| **How concerned are you about your illness?** | | | | | | | |  |  |
| 0 1 | 2 | 3 | 4 | 5 | 6 | 7 | 8 | 9 | 10 |
| not at all |  |  |  |  |  |  |  |  | extremely |
| concerned |  |  |  |  |  |  |  |  | concerned |
| **How well do you feel you understand your illness?** | | | | | | | |  |  |
| 0 1 | 2 | 3 | 4 | 5 | 6 | 7 | 8 | 9 | 10 |
| don't understand |  |  |  |  |  |  |  |  | understand |
| at all |  |  |  |  |  |  |  |  | very clearly |
| **How much does your illness affect you emotionally? (e.g. does it make you angry, scared, upset or depressed?**  0 1 2 3 4 5 6 7 8 9 10  not at all extremely  affected affected  emotionally emotionally | | | | | | | | | |
| **Please list in rank-order the three most important factors that you believe caused your illness. *The most important causes for me:-***  **1.**  **2.**  **3.** | | | | | | | | | |
